# Assessing Cancer Risk Associated with Aquatic Polycyclic Aromatic Hydrocarbon Pollution Reveals Dietary Routes of Exposure and Vulnerable Populations

**DOI:** 10.1155/2018/5610462

**Published:** 2018-09-19

**Authors:** Larisa M. Gearhart-Serna, Nishad Jayasundara, Moises Tacam, Richard Di Giulio, Gayathri R. Devi

**Affiliations:** ^1^Department of Surgery, Division of Surgical Sciences, Duke Cancer Institute, Duke University, Durham, NC, USA; ^2^Department of Pathology, Duke Cancer Institute, Duke University, Durham, NC, USA; ^3^Nicholas School of the Environment, Duke Cancer Institute, Duke University, Durham, NC, USA; ^4^School of Marine Sciences, University of Maine, ME, USA; ^5^Trinity School of Arts and Sciences, Duke Cancer Institute, Duke University, Durham, NC, USA; ^6^Women's Cancer Program, Duke Cancer Institute, Duke University, Durham, NC, USA

## Abstract

Polycyclic aromatic hydrocarbon (PAH) exposure is widespread, and many PAHs are considered carcinogenic. The PAH-contaminated AWI Superfund site in Virginia provides a model for studying a complex PAH mixture and its extrapolation to cancer risk and PAH exposure in the general population. We examined cancer risk at the Superfund site due to sediment-derived PAHs and then evaluated PAH sources in the general population and potentially vulnerable subpopulations upon PAH mixture exposure. The PAH mixture was assessed for potential carcinogenicity using the US EPA's OncoLogic™ ranking tool and the US EPA list of priority PAHs. Cancer risk due to PAH exposure was calculated for Superfund site users and compared to the US EPA assessment. Human intake and health endpoints of PAHs within the mixture were extracted from USEtox® chemical fate database, while mean intake exposure was calculated for U.S. adults for select PAHs using NHANES database urinary biomarkers. Eleven PAH compounds within the mixture were of carcinogenic concern, and seven PAHs conveyed significant excess cancer risk at the Superfund site and in the general population, wherein PAH-contaminated seafood ingestion was a main contributor. Other dietary sources of PAHs derived from PAH-contaminated soil or water could also play a role in total exposure. Vulnerable populations to PAH exposure and coinciding increased cancer risk may include, in addition to smokers, children and non-Hispanic blacks, which is a public health concern.

## 1. Introduction

From prenatal days until death, humans are each subjected to their own variable exposome [1], a sum of individual environmental exposures from their source to their biological and health effects [2–4]. Polycyclic aromatic hydrocarbons (PAHs) are highly carcinogenic, are also considered teratogenic and mutagenic, and may act as endocrine disrupting chemicals (EDCs). PAHs are released from anthropogenic and natural activities. PAH emissions and human exposure originate from sources of incomplete organic fuel combustion, including tobacco smoke, charred or smoked meat, industrial byproduct emissions, forest fires, volcanic eruptions, and even contaminated food [5]. While air emission sources such as tobacco smoke, and subsequent inhalation of volatile PAHs, has dominated analyses of exposure, dietary sources have been suggested as significant contributors to PAH exposure and potential health risks [6–9].

Single PAH chemical studies have been helpful in the research realm for studying toxicity, but realistic exposure to chemicals occurs in mixtures rather than by single chemicals. Therefore, mixture studies are necessary for relevance to actual human exposure. In this study, we model our investigations after previously obtained PAH-contaminated sediment samples derived from a Superfund site Atlantic Wood Industries (AWI) located in Portsmouth, Virginia, along the Southern Branch of the Elizabeth River. Superfund sites are commonly former industrial sites determined by the U.S. Environmental Protection Agency (US EPA) to be candidates for remediation due to contamination by hazardous wastes that pose a significant risk to human and environmental health. The AWI Superfund site was designated in 1990, following several decades of operations as a wood-treating facility. Subsurface soil, groundwater, and sediment contamination with PAHs has been recorded from AWI activities, including wood treatment, storage, and byproduct disposal. The original AWI Superfund site human health and ecological risk assessment identified a variety of chemicals contaminating the land and regions of the Elizabeth River which make up the site, including dioxins, PAHs, and several heavy metals [10]. Of note, extremely high levels of PAHs were measured in Elizabeth River sediments at the site relative to other Chesapeake Bay and worldwide estuarine areas of concern, suggesting that this site represents one of the most highly PAH-contaminated sites in the world [11]. This site provides an opportunity to study a complex PAH mixture that may represent a real-world exposure scenario.

There is a great need to analyze and mitigate risks due to PAH exposure, which has numerous human health consequences. In addition, matrices other than air must be analyzed for their contribution to PAH exposure, for example, soil and water matrices which may have a large impact on dietary PAH exposure. Based on the high level of PAHs and the complexity of the AWI Superfund site mixture, as well as the knowledge that many PAHs are carcinogenic, we postulated that the sediment-derived PAH mixture components would prove to be cancer relevant and confer cancer risk on the users of the Superfund site through various activities (e.g., fishing) relevant to sediment exposure. As this Superfund site mixture is a model complex PAH mixture that may occur in other regions, we further proposed that increased cancer risk and health effects would also be applicable for the wider U.S. population. Utilizing PAH urinary biomarkers from the U.S. CDC National Health and Nutrition Examination Survey (NHANES), a common method utilized successfully to estimate PAH exposure [12–14] and other biomarkers across the country with a statistical weighted approach [15], we have the capabilities to estimate and rank U.S. demographic subpopulations by PAH exposure, targeting those with high exposure for intervention efforts. This concept circles back to our broader hypothesis that PAH mixtures have the potential to increase cancer risk in the general population, especially for vulnerable and highly exposed populations, thus posing significant public health risks.

## 2. Materials and Methods

### 2.1. US EPA OncoLogic™ Carcinogenic Concern Analysis

PAH chemical components of the PAH Superfund mixture were individually run through the OncoLogic™ carcinogenic concern ranking tool [16], which is structure-based. PAH components that were present in the database and contained only a marginal or negligible carcinogenic concern ranking were not included in the results. OncoLogic™ uses structure-activity relationship (SAR) analysis to predict potential carcinogenicity, based upon structural- and biological-based carcinogenicity of various chemical classes. It incorporates both chemical-specific input as well as knowledge-based rules developed by cancer and system development experts based on scientific publications and cancer studies. Carcinogenicity of mixture components were also assessed using the US EPA list of sixteen priority PAHs [17].

### 2.2. Superfund Site Cancer Risk Assessment Analysis

Risk is characterized by both exposure to and the potential human health hazard of a compound. In the original AWI Superfund site Record of Decision (ROD), operable unit 3 (OU3) was designed to account for human health risk due specifically to exposure to sediments on the site [18]. Five target groups of concern were trespassers (adult and child), recreational persons (swimmer/boater/crabber, adult, and child), and workers (i.e., dredge or other heavy equipment operators, spotter, barge worker, and adult). OU3 groups and assumptions are used for this assessment, but only trespassers and recreational adults and children are included in the final cancer risk assessment due to negligible risk calculated for workers wearing personal protective equipment. Seven major PAH contaminants of concern (COCs) in sediment were identified in the original site risk assessment based on calculated cancer risk and hazard indexes, which were benz[a]anthracene, benzo[a]pyrene (BaP), benzo[b]fluoranthene, benzo[k]fluoranthene, chrysene, dibenz[a,h]anthracene, and indeno[1,2,3-c,d]pyrene. These seven COCs were used for our own subsequent cancer risk assessment based on AWI Superfund site sediment oral and dermal exposure. Equations used to calculate cancer risk are as follows:

  Hazard = CSF *∗* ADAF
  Exposure_oral_ = CDI = CS *∗* IR-S *∗* FI *∗* EF *∗* ED *∗* CF *∗* 1/BW *∗* 1/AT
  Exposure_dermal_ = DAD = DA_event_ *∗* EF *∗* ED *∗* EV *∗* SA *∗* 1/BW *∗* 1/AT
  DA_event_ = CS *∗* CF *∗* AF *∗* ABSd

Risk is a function of hazard multiplied by exposure. For acronyms relating to hazard and exposure calculations, please refer to Supplementary Table 1. Cancer risk calculations for the five groups of concern were completed using Monte Carlo analysis described below with the hazard and exposure factors described above and can be found in Supplementary Table 2 [19–24]. This cancer risk only accounts for direct oral or dermal sediment exposure. These cancer risk values were then compared to cancer risk values calculated for exposure to sediment in the original AWI Superfund site risk assessment performed by the US EPA, for the same seven PAH COCs.

### 2.3. Monte Carlo Simulation Analysis

With risk assessment, there is always some degree of uncertainty. To account for this in our cancer risk calculations, we utilized a Monte Carlo simulation method. While the concentration of each PAH COC in sediment as well as their cancer slope factor was held constant, several parameters of exposure were considered a variable. Body weight was considered a variable. For oral exposure, the ingestion rate, exposure frequency, and exposure duration were randomized for simulation using minimum and maximum quantitations in Table 1. For dermal exposure, the exposure frequency, exposure duration, soil to skin adherence factor, and surface area available for contact were randomized for simulation using minimum and maximum quantitations in Table 1. While standardized values for these variables are set based on US EPA exposure factors guidelines and professional judgement, 25% of error rates were factored into each variable to provide minimum and maximum distributions. 10,000 iterations were calculated for each group: trespasser adults, trespasser children, recreational adults, and recreational children. Results are shown as histograms of distribution and descriptive statistics for each group and exposure route.

### 2.4. USEtox® Chemical Fate and Human Health Analysis

Environmental fate and human intake data were pulled for 7 of the 36 PAHs present in the Superfund site PAH mixture from the USEtox® chemical fate modeling results database. These 7 PAHs are the same as the COCs identified in the Superfund site risk assessment. Intake from fresh water, sea water, natural soil, and agricultural soil was summed to reflect a mixture of all 7 PAHs and can be found in Supplementary Table 3. This intake was distributed by exposure route: inhalation, drinking water, produce, meat and dairy, and fish. Human health characterization data were also pulled in the form of excess cancer cases per kg emitted and daily adjusted life years for each of the seven Superfund site sediment PAH COCs and pooled to reflect excess cancer cases due to PAH mixture exposure, dependent on the environmental compartment into which the mixture is emitted.

### 2.5. Human Intake Exposure Analysis

Metabolite concentrations for four select PAHs were obtained from a National Health and Nutrition Examination Survey (NHANES) database report [25]. Concentrations were stratified into groups by gender (male/female), race (non-Hispanic white, non-Hispanic black, and Mexican American), and smoking status (smoker, nonsmoker, and exposed to secondhand smoke in the home). These concentrations were reported as the unadjusted geometric means of the entire population measured from 2003 to 2008. These PAHs are naphthalene, fluorene, phenanthrene, and pyrene, all present in high concentrations in the complex Superfund-derived PAH mixture. Excretion fractions of these PAHs in urine were obtained from the literature [26, 27], as well as standardized daily adult urine output [28] and adult body weight [21]. Using a back-of-the-envelope reverse dosimetry pharmacokinetic equation model described previously [29], we constructed estimated daily intake exposure for these four PAHs: exposure intake rate (*μ*g/kg-day) = (concentration_urine metabolite_ × daily adult urine output × MW_parent_)/(urinary excretion fraction × adult body weight × MW_metabolite_).

## 3. Results and Discussion

### 3.1. Polycyclic Aromatic Hydrocarbons Are Present in Superfund Sediment and Are Cancer Relevant

In order to assess the cancer relevance of our Superfund site PAH mixture, the 36 known PAH chemical components in the mixture [30] were assessed for potential carcinogenicity. Most importantly, we report the cancer relevance of many of the PAH chemicals in the AWI Superfund site sediment mixture based on rankings by the US EPA and the OncoLogic™ database tool. All 16 PAH chemicals on the US EPA list of priority PAHs [17] were present in the AWI sediment mixture. Furthermore, several PAHs within this PAH mixture also ranked at least low or low-moderate carcinogenic concern level by the US EPA OncoLogic™ ranking tool of carcinogenic concern (Table 2). Additionally, there may be potential synergy between PAH compounds with different biological targets. For example, high-affinity PAH cytochrome p450 inhibitors and aryl hydrocarbon receptor (AhR) agonists have previously been found to work together to increase toxicity through the AhR pathway in developmental toxicity models [31, 32]. Thus, we could potentially be underestimating the carcinogenic concern of the mixture as a whole. Taken together, this information reveals the considerable carcinogenic concern of the AWI superfund-derived PAH mixture as a whole.

### 3.2. Seafood PAH Exposure Drives Cancer Risk at the Superfund Site

**Figure 1:**
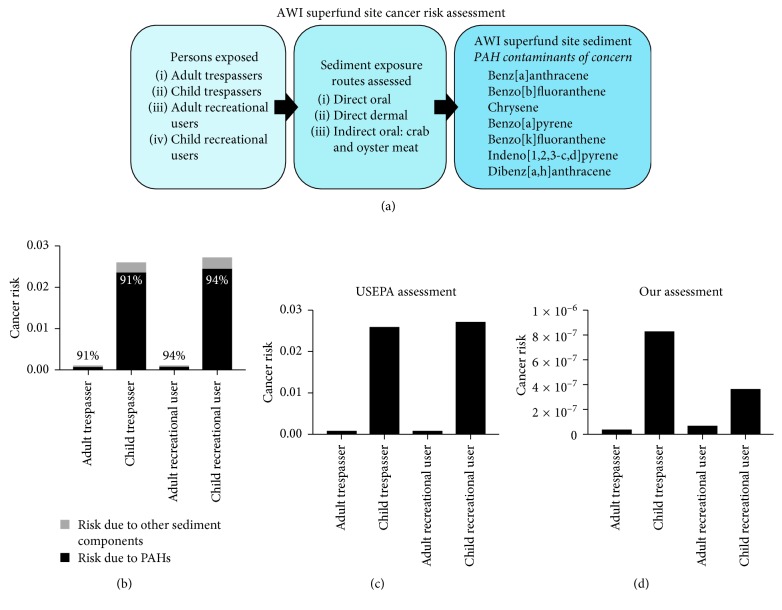
Indirect PAH exposure drives sediment-based cancer risk at the Superfund site. (a) Schematic calculation design of the original Superfund site cancer risk assessment due to sediment exposure, whereas our assessment does not take into account indirect oral exposure. Also includes the seven PAH contaminants of concern in sediment utilized for the cancer risk assessment, both AWI and our assessment. (b) Total cancer risk calculated in the original AWI risk assessment at the Superfund site due to sediment exposure, with the percentage of that cancer risk due to PAH or other contaminant exposure. Cancer risk due to Superfund site sediment exposure as calculated in the original AWI Superfund site risk assessment (c) and as calculated in our direct exposure assessment (d) in this experiment.

**Figure 2:**
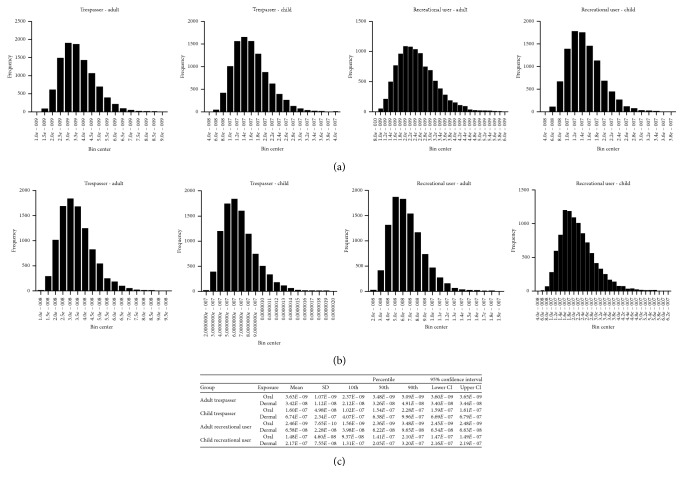
Monte Carlo simulation of cancer risk at the AWI Superfund site. Histograms of cancer risk distribution due to (a) oral exposure and (b) dermal exposure to seven PAH COCs in AWI Superfund site sediment by adult trespassers, child trespassers, adult recreational users, and child recreational users. (c) Descriptive statistics table of cancer risk distributions from Monte Carlo Simulation. COC = contaminant of concern.

The original US EPA AWI Superfund site risk assessment was based on several exposure factors, which accounted for direct sediment ingestion and dermal contact exposure, as well as indirect exposure from eating meat of crab and oyster from the site. From the cancer risks calculated therein, the assessment identified seven PAH contaminants of concern (COCs) that accounted for a majority of the cancer risk (Figure 1(a)). Of the total cancer risk due to the AWI sediment exposure, for all sediment components, over 90% was attributable to the PAH composition of the contaminated sediment for each group of concern, trespasser and recreational user adults and children of the Superfund site (Figure 1(b)). In our study related to the AWI sediment exposure assessment, cancer risks were calculated for each group of concern due to sediment exposure to the seven COCs, utilizing Monte Carlo simulations to estimate cancer risks. We utilized a standard 25% variability on exposure factors such as visits per day to the site and ingestion rates, due to known fluctuation but uncertain range distributions (Table 1). For these simulations, we only calculated risks due to direct oral ingestion or dermal exposure to PAH-contaminated sediments. We observed that our mean cancer risk calculations were orders of magnitude lower than the original US EPA cancer risk assessment to the seven sediment PAH COCs (Figures 1(c) and 1(d)). In both the US EPA and our assessment, children had higher cancer risk than adults due to this AWI Superfund PAH mixture exposure for both trespassers and recreational users of the site. 95% confidence intervals of associated cancer risk revealed reliable risk calculations, with less than 0.03 E–07 range between lower and upper bounds and steep bell curve models when shown by histogram. In addition, oral and dermal exposure simulation distributions for all groups of concern were left-skewed, with larger variability from dermal exposure routes (Figures 2(a)–2(c)). Variability was set at 25%, so we were limited in that risk assessment variables with larger ranges (i.e., body weight, exposure frequency, and body surface area) having larger effects on cancer risk.

While we had complete information on ingestion and dermal risk assessment parameters, we had no data regarding current seafood intake or parameters for the Superfund site. However, the difference in cancer risk between assessments is likely due to our cancer risk assessment not accounting for the indirect exposure to crab and oyster meat from the original risk assessment, since this was the only large difference between the two assessments. When this seafood exposure was taken into account in the original assessment, total cancer risk due to sediment exposure exceeded 10^−4^ for both adult and child trespassers and recreational users of the site, which is the threshold safe cancer risk as defined by the US EPA. Taken together, the results suggest that the sediment-derived PAH mixture obtained for this experiment is of carcinogenic concern in a real-world setting, with contaminated seafood ingestion as the largest contributor to PAH exposure. In translating this knowledge to the broader public, contaminated seafood dietary ingestion could be a significant source of exposure to PAH mixtures.

### 3.3. PAH Intake Exposure Varies by Environmental Compartment Source

**Figure 3:**
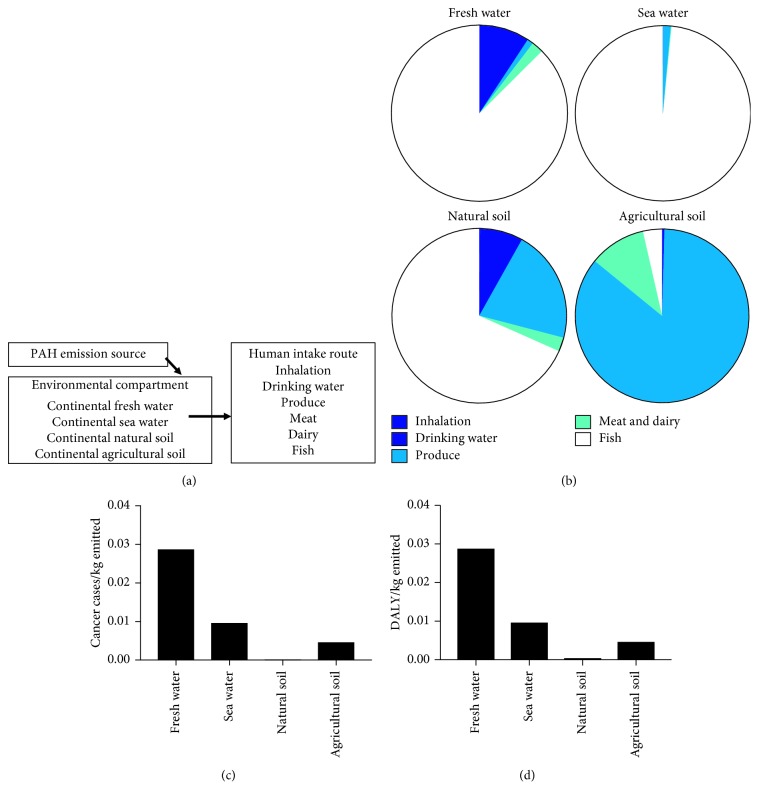
PAH mixture exposure varies in the human population depending on source: (a) chemical fate modeling of PAHs in the environment from emission source to environmental compartment to human intake route, as utilized in the USEtox model; (b) USEtox database distribution of human intake of a mixture of 7 PAH COCs. Human intake is measured by inhalation, drinking water, produce, meat and dairy, or fish, depending on the environmental compartment into which the PAH is emitted; (c) cancer cases and (d) daily adjusted life year (DALY) per kg emitted into different environmental compartments for a PAH mixture containing the seven Superfund site sediment contaminants of concern.

The AWI Superfund site is currently closed for remediation, although adjacent sites are under evaluation for similar PAH contamination levels. In addition, the mixture described previously from Superfund site sediment represents a complex PAH mixture which could be present anywhere in varying amounts, more broadly applicable to the wider human population. We next analyzed the PAH mixture for potential increase in cancer incidence in this larger human population. To do this, we utilized the USEtox® PAH chemical fate database, and the fate schematic from emission to human intake used for the database calculations is shown in Figure 3(a). The PAH mixture considered in these intake analyses included the 7 PAH COCs found in the Superfund sediment PAH mixture.

USEtox® chemical fate database intake factors revealed that PAH exposure in the general human population can occur via multiple routes, and human exposure to PAHs varies depending on where the PAH is emitted. For this study, we focused on soil and water matrices, closely applicable to our Superfund sediment-derived PAH mixture. For example, if the PAH mixture is emitted into continental fresh water, continental sea water, or continental natural soil, the mixture is taken in by humans primarily through fish, which is consistent with the increased cancer risk seen at the Superfund site due to contaminated sediment exposure. PAH mixture intake can also occur through drinking water and produce (Figure 2(b)). Thus, lifestyle and diet factors can substantially impact an individual's exposure to these PAH chemicals.

USEtox® also allows analysis of midpoint and endpoint human health effects due to chemical emission and exposure. Cancer cases and daily adjusted life year (DALY, life lost due to disability or death) per kg emitted of a PAH mixture comprised of the same seven COCs in the Superfund site sediment varied depending on where the PAHs are sourced. The largest cancer risk and DALY came from emission into household indoor air (data not shown), but fresh water also increased cancer cases and DALY based on PAH mixture emissions, followed in magnitude by sea water, agricultural soil, and natural soil (Figure 2(c)). Taken together, the data from this database reveal that a complex PAH mixture similar to our Superfund-derived example can affect human health by potentially increasing cancer cases and DALY, and that exposure to PAHs is widespread and highly variable in terms of source and intake.

### 3.4. Certain Demographic Subpopulations Have Higher PAH Exposure

Perhaps one of the most important considerations when analyzing PAH exposure is to analyze human subpopulations most at risk for exposure, for the greatest translation and impact on public health interventions. We conducted reverse dosimetry modeling to estimate PAH intake exposure based on urinary biomarkers of four PAHs (naphthalene, fluorene, phenanthrene, and pyrene) available in the U.S. CDC NHANES database. Urinary PAH biomarkers have previously been used to estimate exposure, and although PAHs vary seasonally and potentially by the time of urine collection, they provide us with a method to rank subpopulations in terms of exposure rather than just providing exposure quantitations. Additionally, NHANES provides the best available data on PAH biomarkers across a wide range of U.S. adult residents, thus providing a great value to the estimation of exposure across the human population.

**Figure 4:**
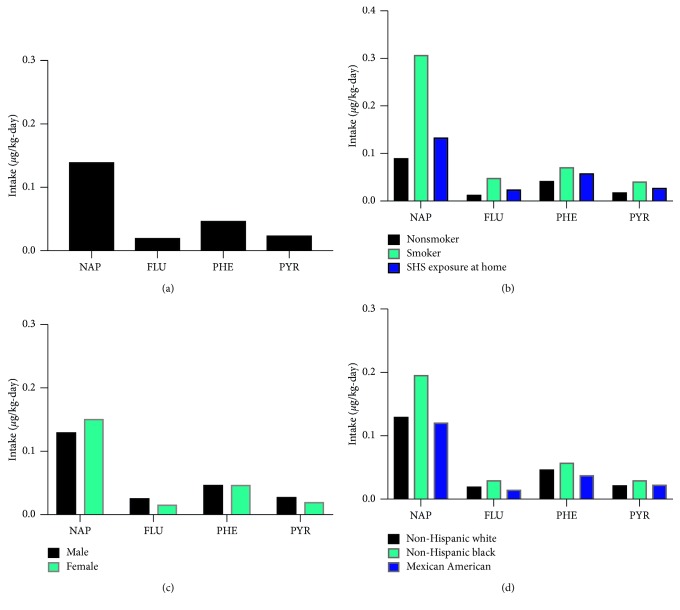
Intake exposure modeling of select PAHs for U.S. adults. Mean intake exposure (μg/kg-day) calculated for (a) all 2003–2008 NHANES participants and stratified by (b) smoking status, (c) gender, or (d) race/ethnicity.

Naphthalene, the simplest and most prolific PAH compound, constituted the largest portion of exposure to the four PAHs (Figure 4(a)). Not surprisingly, when separated by the smoking status, users of tobacco smoke had much higher exposure than nonsmokers, and those exposed to secondhand smoke were also exposed more than nonsmokers (Figure 4(b)). Once stratified by demographics factors gender and race/ethnicity, neither males nor females had significantly higher exposure to any of the four PAHs (Figure 4(c)), but non-Hispanic blacks had higher exposure for all four PAHs than other populations (Figure 4(d)). These analyses do not take into account subgroup interactions, for example, racial/ethnic groups that may be more highly exposed to PAHs when also smokers. However, our findings speak to the potential existence of vulnerable populations to PAH exposure, and the toxic effects included therein, including increased cancer risk. These subpopulations may be targeted in future studies and intervention efforts to reduce exposure.

## 4. Conclusion

In sum, we see increased cancer risk for children at the Superfund site, and in general, the majority of cancer risk comes from seven PAH COCs and contaminated seafood ingestion. We also see that this exposure can vary greatly in the general human population depending on where the PAHs are emitted and how they are taken in, and that non-Hispanic black subpopulations in addition to smokers may be especially vulnerable to toxic effects and cancer risk due to PAH mixture exposure. This has major implications for public health and chemical exposure disparities.

## Figures and Tables

**Table 1:** Monte Carlo simulation variables and distributions for cancer risk assessment due to oral and dermal sediment exposure at the AWI Superfund site.

Parameter	Symbol	Units	Distribution-AT	Distribution-CT	Distribution-AR	Distribution-CR
Concentration of 7 PAHs in sediment	CS	mg/kg	0.283	0.283	0.283	0.283
Average time-Cancer	AT	days	25550	25550	25550	25550
Age-dependent adjustment factor	ADAF	Unitless	1	10	1	10
Conversion factor	CF	Unitless	0.000001	0.000001	0.000001	0.000001
Body weight	BW	kg	80 ± 20	15 ± 3.75	80 ± 20	15 ± 3.75

*Oral exposure*						
Benzo(a)anthracene cancer slope factor	BaA	mg/kg-day	7.30*E* − 01	7.30*E − *01	7.30*E − *01	7.30*E − *01
Benzo(a)pyrene cancer slope factor	BaP	mg/kg-day	7.30*E + *00	7.30*E + *00	7.30*E + *00	7.30*E + *00
Benzo(b)fluoranthene cancer slope factor	BbF	mg/kg-day	7.30*E − *01	7.30*E − *01	7.30*E − *01	7.30*E − *01
Benzo(k)fluoranthene cancer slope factor	BkF	mg/kg-day	7.30*E − *02	7.30*E − *02	7.30*E − *02	7.30*E − *02
Chrysene cancer slope factor	Chr	mg/kg-day	7.30*E − *03	7.30*E − *03	7.30*E − *03	7.30*E − *03
Dibenz(a,h)anthracene cancer slope factor	Dib	mg/kg-day	7.30*E + *00	7.30*E + *00	7.30*E + *00	7.30*E + *00
Indeno(1,2,3-c,d)pyrene cancer slope factor	Ind	mg/kg-day	7.30*E − *01	7.30*E − *01	7.30*E − *01	7.30*E − *01
Exposure frequency	EF	days/year	40 ± 10	52 ± 13	32 ± 8	48 ± 12
Exposure duration	ED	years	20 ± 5	6 ± 1.5	20 ± 5	6 ± 1.5
Ingestion rate-sediment	IR-S	mg/day	20 ± 5	50 ± 12.5	20 ± 5	50 ± 12.5

*Dermal exposure*						
Benzo(a)anthracene cancer slope factor	BaA	mg/kg-day	2.50*E + *00	2.50*E + *00	2.50*E + *00	2.50*E + *00
Benzo(a)pyrene cancer slope factor	BaP	mg/kg-day	2.50*E + *01	2.50*E + *01	2.50*E + *01	2.50*E + *01
Benzo(b)fluoranthene cancer slope factor	BbF	mg/kg-day	2.50*E + *00	2.50*E + *00	2.50*E + *00	2.50*E + *00
Benzo(k)fluoranthene cancer slope factor	BkF	mg/kg-day	2.50*E − *01	2.50*E − *01	2.50*E − *01	2.50*E − *01
Chrysene cancer slope factor	Chr	mg/kg-day	2.50*E − *02	2.50*E − *02	2.50*E − *02	2.50*E − *02
Dibenz(a,h)anthracene cancer slope factor	Dib	mg/kg-day	2.50*E + *01	2.50*E + *01	2.50*E + *01	2.50*E + *01
Indeno(1,2,3-c,d)pyrene cancer slope factor	Ind	mg/kg-day	2.50*E + *00	2.50*E + *00	2.50*E + *00	2.50*E + *00
Exposure frequency	EF	days/year	40 ± 10	52 ± 13	32 ± 8	48 ± 12
Exposure duration	ED	years	20 ± 5	6 ± 1.5	20 ± 5	6 ± 1.5
Soil to skin adherence factor	AF	mg/cm^2^-day	0.07 ± 0.0175	0.2 ± 0.05	0.2 ± 0.05	0.07 ± 0.0175
Dermal absorption fraction	ABSd	Unitless	0.13	0.13	0.13	0.13
Skin surface area available for contact	SA	cm^2^	6032 ± 1508	2373 ± 593.25	6032 ± 1508	2373 ± 593.25

AT = adult trespasser; CT = child trespasser; AR = adult recreational user; CR = child recreational user.

**Table 2:** Cancer-relevant polycyclic aromatic hydrocarbons are present in Superfund sediment, as determined by US EPA priority PAHs list and OncoLogic™ carcinogenicity ranking tool.

	Concentration^*∗*^ (ng/mL)	Standard deviation (ng/mL)	US EPA priority PAH	OncoLogic™ carcinogenic concern level
Naphthalene	1617.2	341.2	+	
Phenanthrene	597.1	171.7	+	Low
Fluoranthene	422.6	35.7	+	
Acenaphthene	404.7	96.4	+	Low
Fluorene	321.2	188.5	+	
Pyrene	288.0	21.4	+	Low
Carbazole	246.0	60.1		
Dibenzofuran	207.7	82.9		
1-Methylnaphthalene	161.5	31.7		
Benz(a)anthracene	77.6	7.8	+	Low-moderate
Anthracene	73.1	12.1	+	
Benzo(b)fluoranthene	66.1	4.8	+	Moderate-high
Dibenzothiophene	63.4	49.8		
Chrysene	62.0	4.7	+	
1,2-Benzofluorene	46.5	4.1		
2,6-Dimethylnaphthalene	44.7	39.4		
Benzo(a)pyrene	44.3	2.4	+	High
Retene	43.8	3.6		
2-Methylphenanthrene	39.8	10.7		
Benzo(e)pyrene	31.2	7.3		
Benzo(k)fluoranthene	26.6	1.7	+	Low-moderate
1-Methylphenanthrene	20.5	3.1		
Acenaphthylene	17.0	8.5	+	
Benzo(g,h,i)perylene	14.7	1.3	+	
Picene	14.2	1.2		Moderate
3,4-Benzofluorene	9.0	3.0		
Perylene	8.8	0.8		Low
Benzo(a)fluoranthene	6.9	0.7		Low-moderate
Dibenz(a,l)pyrene	6.2	0.5		High
Indeno(1,2,3-c,d)pyrene	3.6	0.5	+	Moderate
Benzo(b)chrysene	3.5	0.4		
Dibenz(a,j)anthracene	2.8	0.6		Moderate
Dibenz(a,h)anthracene	2.8	0.6	+	High
Benzo(c)phenanthrene	2.4	0.3		Low-moderate
3-Methylcholanthrene	0.0	0.0		

^*∗*^Concentrations determined by mass spectrometry in previous study [22].

## Data Availability

Data collected for this study are freely available in US EPA reports on Atlantic Wood Industries, the USEtox database, and other public domains.

## Supplementary Materials

Supplementary Materials Supplementary Table 1: AWI Superfund site cancer risk, hazard, and exposure acronym explanations, values, and data sources. Supplementary Table 2: raw exposure and cancer risk calculations for users of the AWI Superfund site by contaminant of concern.
